# A Preliminary Investigation of the Running Digit Span As a Test of Working Memory

**DOI:** 10.3233/BEN-2008-0212

**Published:** 2009-06-01

**Authors:** Marjan Jahanshahi, T. Saleem, Aileen K. Ho, R. Fuller, Georg Dirnberger

**Affiliations:** ^1^Cognitive Motor Neuroscience GroupSobell Department of Motor Neuroscience & Movement DisordersUCL Institute of NeurologyThe National Hospital for Neurology & NeurosurgeryQueen SquareLondonUK; ^2^Centre for Palliative CareKings CollegeLondonUK; ^3^Department of PsychologySchool of Psychology and Clinical Language SciencesUniversity of ReadingReadingUK; ^4^Maryland Psychiatric Research CenterUniversity of MarylandBaltimoreMDUSA; ^5^Department of NeurologyDivision of NeurorehabilitationUniversity of ViennaViennaAustria

**Keywords:** Running span, episodic memory, executive function, monitoring, updating

## Abstract

The objective of this study was to compare performance on different versions of the running span task, and to examine the relationship between task performance and tests of episodic memory and executive function. We found that the average capacity of the running span was approximately 4 digits, and at long sequence lengths, performance was no longer affected by varying the running span window. Both episodic and executive function measures correlated with short and long running spans, suggesting that a simple dissociation between immediate memory and executive processes in short and long running digit span tasks may not be warranted.

## 1. Introduction

The term working memory was introduced by Baddeley and Hitch [1], to replace the older concept of short-term memory. It refers to a brain system that provides temporary storage and manipulation of information necessary for such complex cognitive tasks such as language comprehension, learning and reasoning [2]. This capacity to access and hold information 'on-line' was attributed to the prefrontal cortex. Evidence from a variety of sources, animal lesions studies [21,22], studies of patients with prefrontal lesions [14,19,25,35], and from PET activation studies [9,11,12,23,24] has more specifically shown that it is the dorsolateral prefrontal cortex (DLPFC, Walker's area 46 and Brodmann's area 9) which is involved in tasks engaging working memory. Although a variety of different tasks have been used across these studies, the common feature is that they involved monitoring of behaviour, holding information 'on-line' and intrinsic response generation.

The running span or memory paradigm was initially used by Pollack, Johnson and Knaff [26]. This is a task in which the role of the central executive is believed to be distinguished from that of the slave systems. The task requires subjects to watch strings of consonants of unknown length or to listen to a series of digits and then to recall serially a specific number of recent items (fixed partial recall). The running span task requires flexibility of information processing and a progressive shift of attention, that is, discarding some items while new ones are registered. Morris and Jones [16] showed that the running span task requires two independent mechanisms: the phonological loop (phonological store and articulatory rehearsal process) and the central executive.

ISSN 0953-4180/08/$17.00 © 2008 – IOS Press and the authors. All rights reserved

Process analysis of the running digit span test suggests that performance requires the following:

1. Holding the first few items presented in memory;
2. Continuous monitoring of incoming information;
3. Repeated updating of information by appending the newest item and discarding the old item from the target string, everytime another item is presented

All of these processes are attention-demanding. While the phonological loop or visuospatial sketchpad enable the on-line temporary store of the auditorily or visually presented digits in working memory, the central executive must monitor the continuous stream of incoming information and also actively update the on line contents every time an additional supra-span item is presented. This continual refreshing and shifting of the on-line working memory frame is the hallmark of the dynamic running span test. Consequently, there are considerable performance costs for longer sequences [20,26], faster presentation rates [5,26], uncertain length of sequences [8,26], the presence of competing distractor information [32], and also increasing age [32].

Current interest in the fractionation of executive function, and indeed the question of the unitary or diversity of these subcomponents [10,15,33] has renewed interest in the running span task as a measure of working memory 'updating'. Our understanding of various parameters within this task and the extent to which episodic memory and executive function influence performance on this task is still limited. In the present study, our aim was to:

(i) Investigate the effect of list length and report span length on the running digit span task concommitantly (Experiment1), and to replicate key findings in a larger study (Experiment 2).
(ii) Examine how performance on the running span task relates to measures of episodic memory and executive function (Experiment 2)

There are different hypotheses regarding the mechanism of the running span task [5], the most popular being the high effort on-line rehearsal and active updating of material during presentation [27,28]. A more low effort passive transfer of material after presentation has also been suggested [7]. We predicted that if updating is key, sequence length will be important, and short report span lengths with frequent updating may be more strongly associated with tests of executive function, while the longer version which is close to maximal memory span capacity will show a greater correlation with immediate memory measures.

## 2. Experiment 1

In the running span paradigm, a variety of list lengths are used in order to introduce uncertainty of list length, so that participants have to continually update the requisite "report span" on-line. This unpredictability of overall list length effectively stops participants from resorting to recall strategies which can be applied to a known list length [8,26]. The running digit "report span length" refers to the number of items from the end of the list that the participants have to recall, for example the last 3, 5 or 7 items of the list. Performance on the task with list length as a variable of interest in its own right has not yet been investigated in conjunction with running digit "report span length". This is interesting because the length of the entire sequence in which the updating window is gradually shifted across time may contribute to the overall task load and complexity. Practically, this will also be useful in understanding the impact of altering these two key parameters (overall sequence length, and running digit span report) in various versions of the task, as this is presently unclear.

## 3. Methods

### *3.1. Participants*

The participants were ten (4 male, 6 female) healthy normals who had no previous history of neurological, psychiatric, physical illness, head injury, or alcohol or drug abuse and were not taking any medication at the time of assessment. The mean age was 30.70 years (sd = 12.38, range = 19 to 59).

### *3.2. Design and procedure*

A within-subject repeated measures design was used. Participants completed the running digit span task [26] according to standard instructions. The running digit span task used was constructed according to the details provided by Talland [31]. Participants were presented with sequences of 8, 15 or 18 numbers between 1 and 9 randomly presented on a screen at the rate of one digit per second. Across 3 separate blocks of 6 trials each (2 trials of lengths of 8, 15 or 18 digits), the subject's instructions were to remember the last 3 (rundig3), 5 (rundig5), or 7 (rundig7) numbers of a sequence. On each trial, the participantt knew the number of digits that had to be retained and recalled at the end of the sequence, but was not informed about the length of

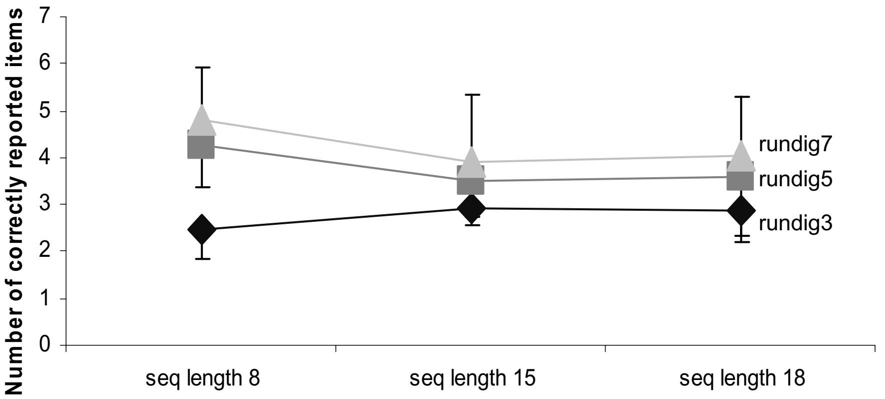

Fig. 1. Running digit span score of participants when asked to recall the last three (rundig3), five (rundig5) and seven (rundig7) digits in sequences of different lengths (8, 15, 18 numbers).

the sequence. Participants were required to recall the last 3, 5 or 7 items of a series verbally and in the correct order. A fixed order of blocks was used, and all participants started with the block requiring recall of the last 3 items in a sequence and finished with the block of trials requiring retention and recall of the last 7 numbers. The score was the total number of items correctly recalled in the correct serial position. Separate subscores were obtained for recalling the last 3, 5 or 7 items for sequence lengths of 8, 15 and 18 digits.

## 4. Results

A two-way ANOVA with factors of Running digit span (3, 5, 7) and Sequence length (8, 15, 18) was performed. There was a significant main effect of Running digit span (F(2,18) = 13.09, $p < 0.05$), and a significant Running digit span x Sequence length interaction F(4,36) = 2.65, $p < 0.05$). The main effect of Sequence length was not significant (F(2,18) = 1.16, $p > 0.05$). Figure 1 shows this interaction, the average number of items correctly recalled by the participants is shown for the running span lists with 8, 15 or 18 items when asked to recall (in serial order) the last 3, 5 and 7 items. This interaction was followed-up by three one-way ANOVAs examining the effect of Running digit span (3, 5, 7) at each sequence length. There was a significant effect for sequence length 8 (F(2,18) = 22.14, $p < 0.05$), but not for longer sequence lengths of 15 and 18 (F(2,18) = 2.69, $p > 0.05$ and F (2,18) = 3.50, $p > 0.05$) respectively). Paired t-tests showed that for sequence lengths of 8 digits, there was a significant difference between running digit spans of 3 and 5 (t (9) = 6.91, $p < 0.05$), and between 3 and 7 (t(9) = 4.92, $p < 0.05$), but not between 5 and 7 (t(9) = 1.63, $p > 0.05$).

## 5. Discussion

In Experiment 1, when overall sequence lengths were 15 or 18 digits long, there was no difference in the number of digits correctly reported regardless of running span instructions , that is the requirement to recall the last 3, 5, or 7 items. This suggests that once working memory capacity is clearly exceeded, running span parameters specifying the length of the updating window do not significantly affect recall performance. When the overall sequence length is 8 digits, and therefore within working memory span, then the updating window or running span required influences performance. Participants appeared to be equally good at reporting the last 3 digits regardless of the length of digits presented. Correct serial reporting of items in a running span task increases up to full capacity of approximately 4 digits when participants are instructed to recall the last 5 digits rather than just 3. However, there was no further increase in correct report when recall instructions were increased to the last 7 digits, suggesting that serial running span report plateaus at about 4 digits, corroborating the figure previously reported [7,26].

## 6. Experiment 2

While central resources are clearly implicated in successful performance of the running span task, their specific role is not clearly understood. Furthermore, this task has been relatively under-used compared to other tests known to tap the central executive. Therefore a concurrent examination of the running digit span task and well-established tests of episodic memory and executive function would be useful in understanding the nature of the executive contribution to the process of maintaining an "on line" representation of items in memory. A comprison of several versions of the test with different report span lengths will also yield important information when formulating a standard version with clinical use in mind. The aim of Experiment 2 was to:

1. Replicate Experiment 1 in a larger sample, and to compare performance on 3 versions of the running span test as defined by report span required, collapsing across length of digit sequences used.
2. Examine how performance on 3 versions of the running span test relate to episodic memory (digit span forward, Rey Auditory Verbal Learning test) and to tests of executive function (digit span backwards, verbal fluency, random number generation)

We predicted that central resources would be more involved in the shortest version of the test where only the last three digits are reported due to the need to update the online store of items more frequently; whereas the load on the phonological loop should be low due to the short span. The converse would apply for the longest version of the task; while reporting seven digits should require a longer working memory span and involve greater immediate memory load, the number of updates required by central executive processing would be less due to the longer span. On this basis, it might be expected that the short version with frequent updating may be more strongly associated with tests of executive function, while the longer version which is close to maximal memory span capacity will show a greater correlation with immediate memory measures.

## 7. Method

### *7.1. Participants*

The participants were twenty one (11 male, 10 female) healthy normals who had no previous history of neurological, psychiatric, other physical illness, head injury, alcohol or drug abuse and were not taking any medication at the time of assessment. They had not previously participated in Experiment 1. Their mean age was 44.05 years (sd = 15.62, range = 23 to 75). All (bar one participant) were right-handed (mean handedness score = 89.02, sd = 14.58, range = 45 to 100), and their mean verbal IQ as estimated by the National Adult Reading Test was 117.5 (sd = 6.58, range = 103 to 126).

### *7.2. Design and procedure*

A within subject repeated measures design was used. For this preliminary study, we selected more common standardised measures of executive function and short-term memory in clinical use, as well as random number generation because it is a data-rich task which can be quickly administered. All participants completed a Handedness Inventory [18] and completed the following tests.

1. The Rey Auditory Verbal Learning Test (RAVLT) [29]. Participants listen to a list of 15 words, which the examiner reads aloud at the rate of one word per second, before attempting to recall the words remembered, in any order. To assess learning across trials, this procedure is repeated five times. Delayed recall and delayed auditory recognition of the words was examined 20 minutes later. The number of correct recall on each trial is noted, with the maximum being 15.
2. Random Number Generation (RNG) [3]. Participants are asked to verbally generate numbers from 1 to 9 in a random fahsion. The analogy of picking out numbers out of a hat, with replacement, was used to explain the concept of randomness. Performance was paced with a flashing 1 cm $\times$ 1 cm white square presented on the black screen at the rate of once every 2 seconds. Subjects were instructed to synchronize their RNG responses with the visual pacer, and they produced (100) responses until asked to stop. The measures of randomness analysed were as follows:
   - i) The chi-squared (CHI) statistic, which is a zero order measure of the frequency distribution which may give some index of response preference or bias.
   - ii) Repetitions (REP), which measures the number of times the individual repeats the same item on successive trials. For example, 7-7 counts as 1 repeat, and 1-1-1 counts as 2 repeats.

iii) RNG index, which is a first order measure which reflects any disproportion of digrams in the matrix adjusted for disproportions in the marginal cell frequencies. It varies between 0 and 1, and the higher the index the less random the series is.

iv) The digram matrix looks at the frequency with which each item in the set is followed by each of the possible items. For example, A is paired with B, B is paired with C and so on. In a series with a set size of n (9 in our case), there are $n^2$(81) possible pairings. We obtained several first order measures reflecting the digram matrix. Digrams Achieved (DIG), is the number of non-empty cells in the digram matrix. Digram Repetition Index (DRI) is the sum of all non-empty cell frequencies in the digram matrix, minus one.

v) Unique Triplets (TRI), which is a second order measure. There are N-2 , that is 98, triplets in a series of 100 responses. The number of triplets that are unique are then counted. The fewer the number of unique triplets, the greater the tendency to repeat certain stereotyped second order runs.

vi) Count Scores, which are measures of seriation. We obtained count scores using the general method of Spatt and Goldenberg [30]. Count Score 1 (CS1), measures the tendency to count in ascending or descending series in steps of 1. For example, 1-2-3 or 8-7-6-5-4. All count scores take the length of the series into account. In calculating the count scores, the sequence length is squared to give higher weights to runs of longer sequences. Therefore, these two examples would result in respective count scores of 4 ($\text{CS1} = 2^2$) and 16 ($\text{CS1} = 4^2$). Count Score 2 (CS2), measures the tendency to count in ascending or descending series in steps of 2, for example 2-4-6-8 or 7-5-3-1. Individuals may have count scores that are lower than predicted from a random series if they are avoiding particular counting tendencies or they may have a score which is too high if they are unable to suppress particular counting tendencies.

vii) Gap Score (GAP) is a measure of cycling through the set of 9 items. To obtain this measure, the gap between every occurrence of 1 is noted, then gaps between every occurrence of 2 is noted and so on and the median is calculated. Higher scores indicate that the individual cycles through the series of 9 numbers in a regular fashion so that the numbers are too evenly spread out.

3. Verbal fluency. For the letter fluency task, participants were required to orally generate as many words (excluding proper nouns, numbers and variants of the same root word) which began with the letters ‘f’, ‘a’, and ‘s’ for 60 seconds each [4]. For the category fluency task, participants were required to name as many animals as they could within 60 seconds [6]. All responses were recorded verbatim. The number of correct words (excluding repetitions or incorrect words) for each letter and for the category fluency task were determined.
4. Digit span subtest of the Wechsler Adult Intelligence Scale Revised (WAIS-R) [34], including backward as well as forward span was completed.
5. For the running digit span task, participants were presented with sequences of 8, 15 or 18 numbers between 1 and 9 randomly presented on a screen at the rate of one digit per second. Across 3 separate blocks of 6 trials each (2 trials of lengths of 8, 15 or 18 digits), the subject’s instructions were to remember the last 3 (rundig3), 5 (rundig5), or 7 (rundig7) numbers of a sequence. On each trial, the subject knew the number of digits that had to be retained and recalled at the end of the sequence, but was not informed about the length of the sequence. Participants were required to recall the last 3, 5 or 7 items of a series verbally and in the correct order. A fixed order of blocks was used, and all subjects started with the block requiring recall of the last 3 items in a sequence and finished with the block of trials requiring retention and recall of the last 7 numbers. The score was the total number of items correctly recalled in the correct serial position. Separate subscores were obtained for recalling the last 3, 5 or 7 items for sequence lengths of 8, 15 and 18 digits.

## 8. Results

Figure 2 shows the average number of items in the running span (i.e., rundig score) of participants when asked to recall (in serial order) the last 3, 5 and 7 items of a series of digits. Paired-samples t-tests showed

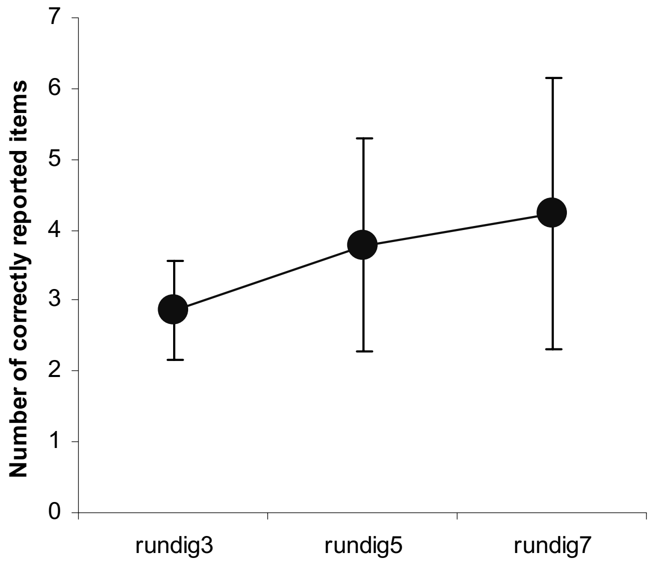

Fig. 2. Running digit span score for recall of the last three (rundig3), five (rundig5) and seven (rundig7) digits.

Table 1
Mean and standard deviation scores on the measures of episodic memory and executive function. Spearman's correlations between running span performance and tests of episodic memory and executive function are also presented

| | Mean (Standard devation) | Rundig3 | Rundig5 | Rundig7 |
|---|---|---|---|---|
| Episodic memory | | | | |
| Digit span forward | 9.7 (2.4) | 0.53* | 0.37 | 0.65** |
| RAVLT total immediate recall | 56.3 (15.7) | 0.11 | 0.08 | 0.02 |
| RAVLT delayed recall | 12.6 (2.8) | 0.21 | 0.26 | 0.48* |
| RAVLT recognition | 14.8 (0.4) | −0.21 | −0.02 | 0.17 |
| Executive function | | | | |
| Digit span backward | 9.3 (2.3) | 0.10 | 0.64** | 0.79** |
| Phonemic fluency | 17.9 (4.6) | 0.05 | 0.26 | 0.16 |
| Category fluency | 19.2 (3.9) | 0.14 | −0.10 | 0.11 |
| RNG–CHI | 4.9 (5.4) | −0.07 | 0.09 | 0.43 |
| RNG–REP | 0.5 (1.2) | −0.07 | 0.31 | 0.41 |
| RNG index | 0.3 (0.04) | −0.64** | −0.01 | 0.05 |
| RNG–DIG | 52.4 (3.0) | 0.47* | −0.10 | −0.35 |
| RNG–DRI | 46.6 (3.0) | −0.47* | 0.10 | 0.35 |
| RNG–TRI | 86.5 (3.6) | 0.53* | −0.14 | −0.26 |
| RNG–CS1 | 30.1 (17.2) | −0.29 | −0.07 | −0.14 |
| RNG–CS2 | 39.9 (11.9) | −0.36 | −0.11 | −0.01 |
| RNG–GAP | 7.9 (0.5) | 0.22 | 0.11 | −0.09 |

**indicates $p < 0.001$, * indicates $p < 0.05$.

that the running digit span for the rundig5 task (3.78 $\pm$ 1.30 items) was significantly longer ($t(20) = 3.49$, $p < 0.05$) than for the rundig3 task (2.86 $\pm$ 0.45 items). There was no significant differences between rundig7 and rundig5 ($t(18) = 1.44$, $p > 0.05$).

The relationship between running digit span performance and measures of episodic memory and executive function were explored using Spearman's correlation coefficients (see Table 1). Adjustments for multiple comparisons were not made because the overall pattern of correlational relationships was primarily of interest.

Among tasks that tapped episodic memory, higher digit span forward score was significantly associated with better running span performance for rundig3 and rundig7, and marginally for rundig5 ($p = 0.010$). Greater delayed recall was significantly correlated with better performance only for rundig7.

On executive function tasks, higher digit span backward score was strongly and significantly associated with better running span performance for longer spans (rundig5 and rundig7). Better performance on the RNG task (as indexed by lower RNG index, higher DIG and TRI scores, and lower DRI score) was significantly correlated better performance on the shortest running span task (rundig3).

When examining Spearman's correlations between running digit span performance and participants' demographic characteristics, there was a significant negative correlation between age and performance on the longest (rundig7) span ($r_s{}^2 = -0.51$, $p < 0.05$), such that older participants were disadvantaged. There were no significant correlations between estimated verbal IQ and running span performance.

## 9. Discussion

Experiment 2, successfully replicated Experiment 1 in a larger sample, confirming the maximal serial running span report of 4 digits. Specifically, it was found that participants were indeed able to significantly increase the number of digits correctly reported by about 1 digit, when the last 5 digits rather than 3 were required. However, there was no further significant increase when the last 7 digits were required, and the average number of digits correctly reported was maximally 4.23.

### *9.1. Association with measures of episodic memory*

Forward digit span was significantly associated with performance on two out of the three running digit span lengths examined, i.e. the shortest (rundig3) and the longest (rundig7), and there was a trend for association with the midlength condition (rundig5). This pattern of results was not predicted, as it was considered that the load on the phonological loop would be low for rundig3, but high for rundig7. The findings suggest the importance of very immediate memory capacity for on-line maintenance of all spans examined here, perhaps because they all fall within immediate memory span limits and there does not appear to be any differentiation between the shortest and longest of these. Contrary to prediction, the longest span length of seven digits did not require any greater immediate memory capacity than the three digits. Immediate recall and delayed recognition were not significantly correlated with any of the running digit span tasks. However, delayed recall, arguably the most demanding of the episodic memory measures used, showed a high and significant correlation with rundig7, the longest span length used which was at the upper end of normal span capacity. These findings suggest that simple immediate memory is important for running digit span tasks in general, and not just for longer running span windows. Delayed episodic memory ability is involved only as span length for the running digits task reaches maximal capacity.

### *9.2. Associaton with measures of executive function*

According to prediction, shorter span length (requiring more frequent updates) did show greater association with executive function as measured by performance on most of the random number generation indicies, but not for the verbal fluency tasks or digit span backwards. There was a significant positive relationship between backward digit span and performance on running digit span lengths of 5 and especially 7, indicating a tigher coupling between executive function and the longest span length. This suggests that longer running span lengths may indeed require greater executive processing, and is contrary to Morris and Jones [16] suggestion, that the number of updates required does not affect performance on the running digit span.

## 10. General discussion

The data suggest that a simple dissociation between immediate memory and executive processes in short and long running digit span tasks may not be warranted. Instead, both short and long running span tasks require a certain level of immediate memory contribution, and long spans draw additionally on delayed recall ability. Regarding the contribution of executive processes, longer running spans are likely to be more tightly linked to the executive processing in terms of ability to hold near maximal spans and to retrospectively manipulate this information, hence the longest span showed the strongest correlation with the backward digit span performance. However, executive processes are also implicated in short running spans as many measures on the RNG task correlated significantly with the shortest running span of 3 digits. These RNG indices of executive function are different from digit span backwards, in that they relate to holding and monitoring information for the purpose of prospective selection and inhibition of strategic responses according to task demands [13]. Perhaps the involvement of working memory and executive function in the running span task are not so clearly segregated as hypothesized [16], and different aspects of working memory and executive function are important in performance at different span lengths.

This study suggests that when report span length exceeds the maximum capacity of 4 digits, the sheer difficulty of the task prompts recruitment of further aspects of both memory (i.e. delayed recall) and executive processes (i.e. digit span backwards) in order to retrospectively manipulate information. Therefore, the increased executive processing demands for longer running spans may drive the closer coupling between digit span backward and rundig7 performance. This may occur because the maximal capacity rundig7 task constitutes a difficult 'non-routine' situation where executive intervention by the central executive or supervisory attentional system in Norman and Shallice's model [17] is required. The increased difficulty of such a non-routine condition is also supported by the significant inverse relationship between performance on long running spans and age [32]. In fact, it can be argued that retrieval of delayed episodic information also involves increased executive resources, and so it is possible that executive processes are key and underly the change in performance from shorter to longer running span reports.

For clinical application, the concomitant analysis of several versions of the test suggests that, the performance on the three digit running span task may be a suitable measure of simple immediate memory, and the increment between the three digit and seven digit running span tasks may be a good indication of executive integrity, and its application in more challenging non-routine scenarios. Hence two different performance measures can be derived from this brief language-free quick test.

In summary, this study examined the under-used running digit span task, and found that the hypothesized dissociable contribution of working memory and executive function to this task is not clear cut. While this conclusion is necessarily tentative, due to the modest sample sizes and selective executive function tests employed, replication with other tests of executive function [10,15] and stratification by age [10,33], will be useful to confirm and elaborate on these preliminary findings. While more succeptible to overall intelligence [7], it would also be interesting to examine participant-determined recall performance on the running digit span task as a comparison to the fixed partial recall procedure used here [5]. This study provides some insight into the key role that central executive processes play in this task and the results contribute to placing this less well-known test in the context of more established neuropsychological instruments. It also provides some evidence for which forms of the running digit span test may be particularly useful for wider application in clinical research.

## Acknowledgements

The financial support of the Wellcome Trust (MJ, GD) during the course of this study is gratefully acknowledged.
